# Supplementary material for: Simultaneous estimation of genotype error and uncalled deletion rates in whole genome sequence data
Source: PLoS Genet. 2024 May 24;20(5):e1011297. doi: 10.1371/journal.pgen.1011297 (PMC11156439; doi:10.1371/journal.pgen.1011297)
Supplement: S1 Text — (PDF) [file pgen.1011297.s001.pdf]

Text S1: Derivation of $\widehat{\Pi}_I^{i,j}$

Our estimates for the true parental genotype frequencies $\widehat{\Pi}_I^{i,j}$ in MAF interval I are derived using the estimated pre-deletion parental genotype frequencies $\widehat{\Pi}_{\text{pre},I}^{i,j}$ and the uncalled deletion rate Γ_I. Recall that $\widehat{\Pi}_{\text{pre},I}^{i,j}$ is estimated as the observed proportion of each parental genotype pair in MAF interval I after excluding Mendelian-inconsistent trios.

Note that there are only six possible pre-deletion parental genotype pairs (AA-AA, AA-AB, AA-BB, AB-AB, AB-BB, BB-BB), but there are 15 possible true parental genotype pairs (AA-AA, AA-AB, AA-BB, AA-AD, AA-BD, AB-AB, AB-BB, AB-AD, AB-BD, BB-BB, BB-AD, BB-BD, AD-AD, AD-BD, BD-BD). We estimate $\widehat{\Pi}_I^{i,j}$ by considering the ways in which these 15 genotype pairs can occur through the replacement of one or more alleles with deletions in pre-deletions parental genotype pairs. For example, AA-AD can arise from AA-AA if there is a deletion of any of the four alleles and also from AA-AB if there is a deletion of the B allele. Thus, we have that $\widehat{\Pi}_I^{0,3} = 4\widehat{\Pi}_{\text{pre},I}^{0,0}\Gamma_I(1-2\Gamma_I) + \widehat{\Pi}_{\text{pre},I}^{0,1}\Gamma_I(1-2\Gamma_I)$. We derive all $\widehat{\Pi}_I^{i,j}$ this way:

$$\widehat{\Pi}_I^{0,0} = \widehat{\Pi}_{\text{pre},I}^{0,0}(1-2\Gamma_I)^2$$

$$\widehat{\Pi}_I^{0,1} = \widehat{\Pi}_{\text{pre},I}^{0,1}(1-2\Gamma_I)^2$$

$$\widehat{\Pi}_I^{0,2} = \widehat{\Pi}_{\text{pre},I}^{0,2}(1-2\Gamma_I)^2$$

$$\widehat{\Pi}_I^{0,3} = 4\widehat{\Pi}_{\text{pre},I}^{0,0}\Gamma_I(1-2\Gamma_I) + \widehat{\Pi}_{\text{pre},I}^{0,1}\Gamma_I(1-2\Gamma_I)$$

$$\widehat{\Pi}_I^{0,4} = 2\widehat{\Pi}_{\text{pre},I}^{0,2}\Gamma_I(1-2\Gamma_I) + \widehat{\Pi}_{\text{pre},I}^{0,1}\Gamma_I(1-2\Gamma_I)$$

$$\widehat{\Pi}_I^{1,1} = \widehat{\Pi}_{\text{pre},I}^{1,1}(1-2\Gamma_I)^2$$

$$\widehat{\Pi}_I^{1,2} = \widehat{\Pi}_{\text{pre},I}^{1,2}(1-2\Gamma_I)^2$$

$$\widehat{\Pi}_I^{1,3} = 2\widehat{\Pi}_{\text{pre},I}^{0,1}\Gamma_I(1-2\Gamma_I) + 2\widehat{\Pi}_{\text{pre},I}^{1,1}\Gamma_I(1-2\Gamma_I)$$

$$\widehat{\Pi}_I^{1,4} = 2\widehat{\Pi}_{\text{pre},I}^{1,2}\Gamma_I(1-2\Gamma_I) + 2\widehat{\Pi}_{\text{pre},I}^{1,1}\Gamma_I(1-2\Gamma_I)$$

$$\widehat{\Pi}_I^{2,2} = \widehat{\Pi}_{\text{pre},I}^{2,2}(1 - 2\Gamma_I)^2$$

$$\widehat{\Pi}_I^{2,3} = 2\widehat{\Pi}_{\text{pre},I}^{0,2}\Gamma_I(1 - 2\Gamma_I) + \widehat{\Pi}_{\text{pre},I}^{1,2}\Gamma_I(1 - 2\Gamma_I)$$

$$\widehat{\Pi}_I^{2,4} = 4\widehat{\Pi}_{\text{pre},I}^{2,2}\Gamma_I(1 - 2\Gamma_I) + \widehat{\Pi}_{\text{pre},I}^{1,2}\Gamma_I(1 - 2\Gamma_I)$$

$$\widehat{\Pi}_I^{3,3} = 4\widehat{\Pi}_{\text{pre},I}^{0,0}\Gamma_I^2 + 2\widehat{\Pi}_{\text{pre},I}^{0,2}\Gamma_I^2 + \widehat{\Pi}_{\text{pre},I}^{1,1}\Gamma_I^2$$

$$\widehat{\Pi}_I^{3,4} = 4\widehat{\Pi}_{\text{pre},I}^{0,2}\Gamma_I^2 + 2\widehat{\Pi}_{\text{pre},I}^{0,1}\Gamma_I^2 + 2\widehat{\Pi}_{\text{pre},I}^{1,2}\Gamma_I^2 + 2\widehat{\Pi}_{\text{pre},I}^{1,1}\Gamma_I^2$$

$$\widehat{\Pi}_I^{4,4} = 4\widehat{\Pi}_{\text{pre},I}^{2,2}\Gamma_I^2 + 2\widehat{\Pi}_{\text{pre},I}^{1,2}\Gamma_I^2 + \widehat{\Pi}_{\text{pre},I}^{1,1}\Gamma_I^2.$$
