# Supplementary material for: Simultaneous estimation of genotype error and uncalled deletion rates in whole genome sequence data
Source: PLoS Genet. 2024 May 24;20(5):e1011297. doi: 10.1371/journal.pgen.1011297 (PMC11156439; doi:10.1371/journal.pgen.1011297)
Supplement: S2 Text — (PDF) [file pgen.1011297.s002.pdf]

Text S2: Testing optimization methods

We investigated each optimization method implemented in the optim() function in R (Nelder-Mead, BFGS, CG, L-BFGS-B, and SANN) and found that SANN was the most effective at maximizing the log-likelihood when applying the model to the UK Biobank sequence data. Specifically, we used all five optimization methods (with their default arguments) to fit our model on all 101 MAF intervals and found that SANN produced the highest maximized log-likelihood in 47 MAF intervals, which was the highest count across all of the optimization methods. SANN also produced the highest mean maximized log-likelihood across all 101 MAF intervals.
