# Supplementary figures and images for: Simultaneous estimation of genotype error and uncalled deletion rates in whole genome sequence data
Source: PLoS Genet. 2024 May 24;20(5):e1011297. doi: 10.1371/journal.pgen.1011297 (PMC11156439; doi:10.1371/journal.pgen.1011297)

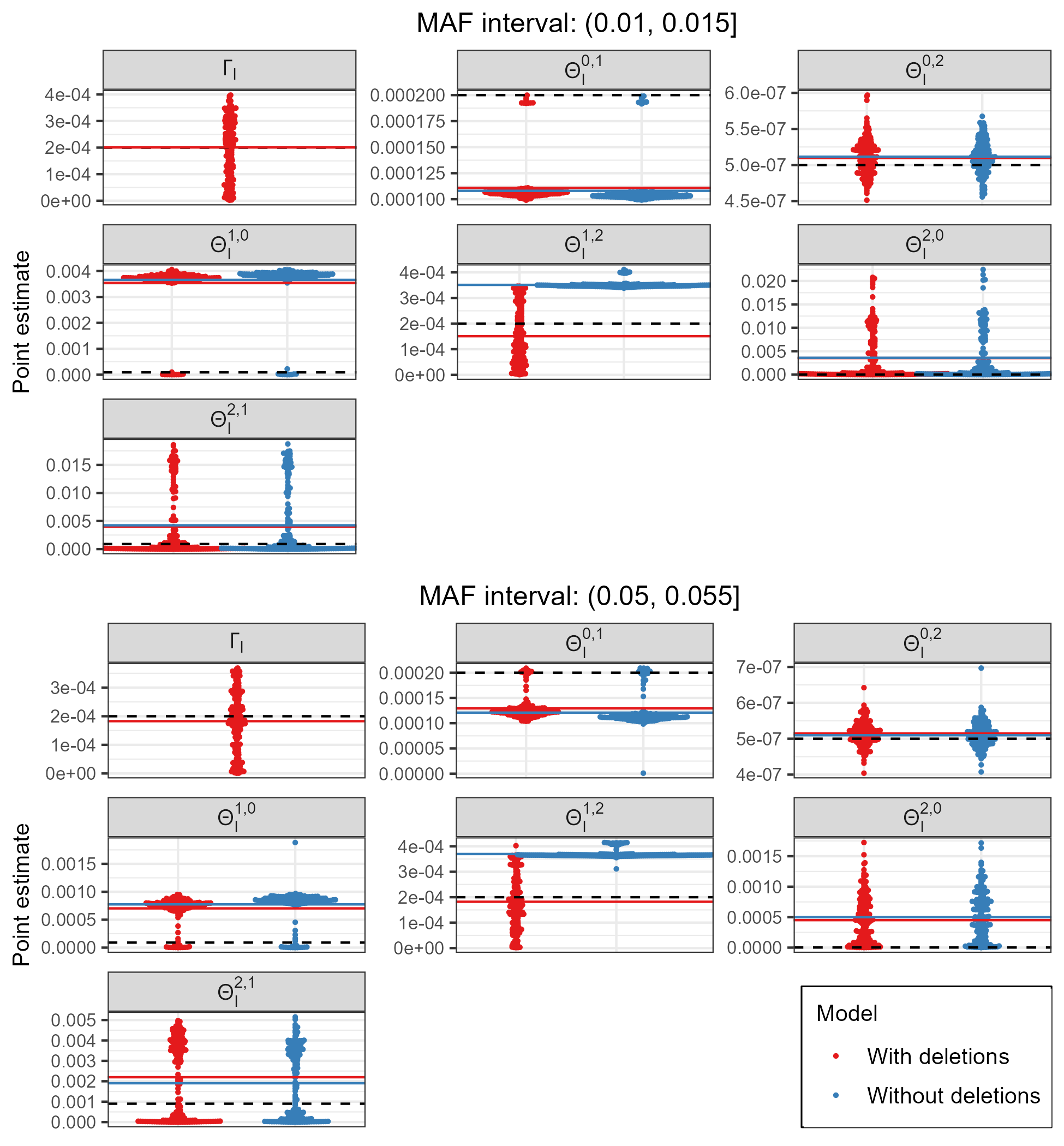

Supplement: S1 Fig — The dashed black line represents the true parameter value from which the observed trio genotypes are simulated. The red and blue lines represent the sample mean of the estimates from the model with and without deletions respectively. (TIF) [file pgen.1011297.s003.tif]

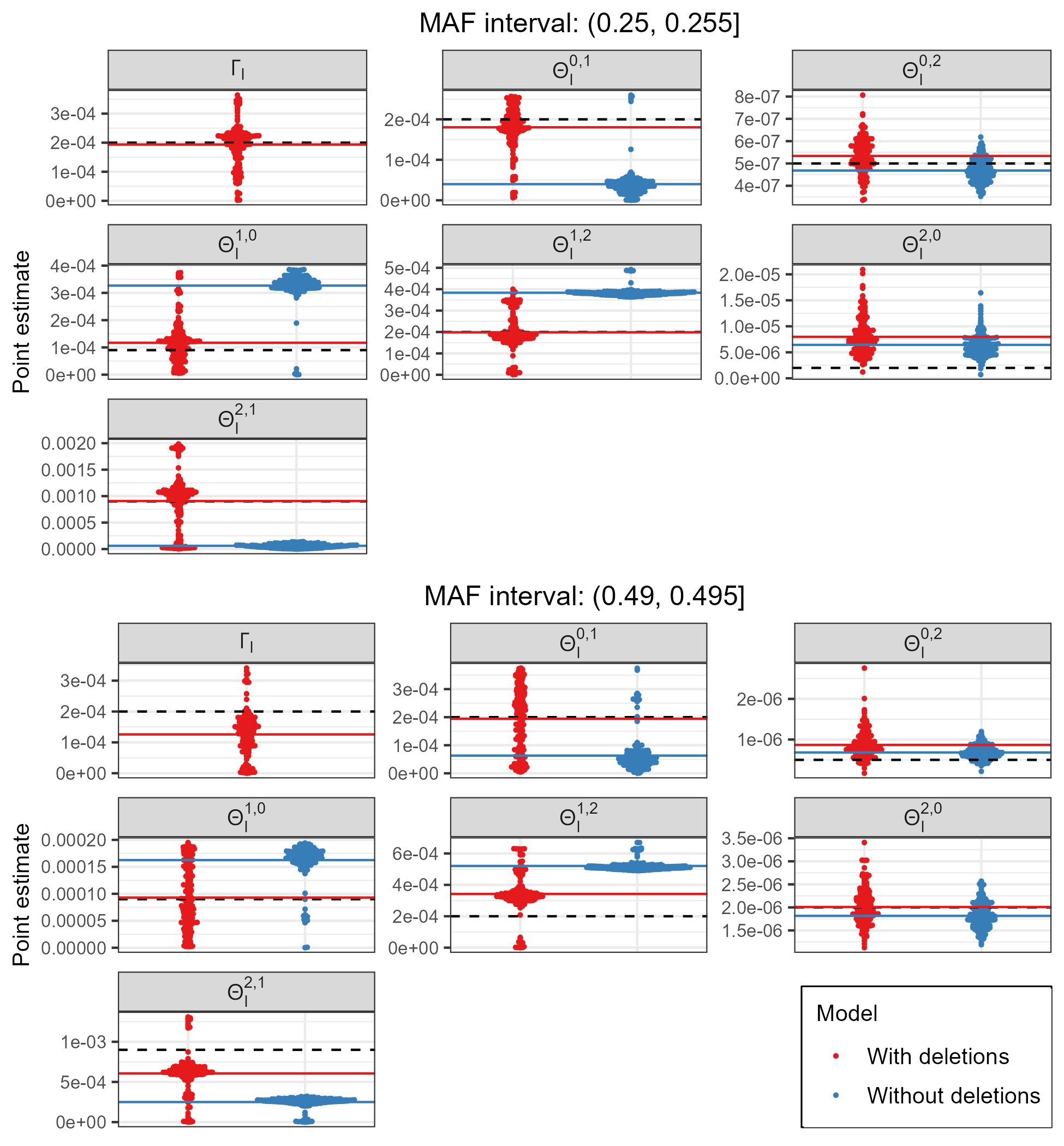

Supplement: S2 Fig — The dashed black line represents the true parameter value from which the observed trio genotypes are simulated. The red and blue lines represent the sample mean of the estimates from the model with and without deletions respectively. (TIF) [file pgen.1011297.s004.tif]
